# Pomegranate Juice Prevents the Formation of Lung Nodules Secondary to Chronic Cigarette Smoke Exposure in an Animal Model

**DOI:** 10.1155/2017/6063201

**Published:** 2017-11-30

**Authors:** Ahmad Husari, Yasmine Hashem, Ghazi Zaatari, Marwan El Sabban

**Affiliations:** ^1^Division of Pulmonary and Critical Care Medicine, Department of Internal Medicine, American University of Beirut, Beirut, Lebanon; ^2^Department of Pathology & Laboratory Medicine, American University of Beirut, Beirut, Lebanon; ^3^Department of Anatomy, Cell Biology and Physiological Sciences, Faculty of Medicine, American University of Beirut, Beirut, Lebanon

## Abstract

**Background:**

Cigarette smoke (CS) induces an oxidative stress, DNA damage, and lung cancer. Pomegranate juice (PJ) possess potent antioxidant activity attributed to its polyphenols. We investigated whether PJ supplementation would prevent the formation of lung nodules, attenuate mitotic activity, and reduce hypoxia-inducible factor-1*α* (HIF-1*α*) expression secondary to CS exposure in an animal model.

**Methods:**

Mice were divided into: Control group, CS group, CS + PJ group, and PJ-only group. CS and CS + PJ were exposed to CS, 5 days per week, for a total of 5 months. Animals were then housed for additional four months. CS + PJ and PJ groups received PJ throughout the experiment period while others received placebo. At the end of the experiment, the incidence of lung nodules was assessed by (1) histological analysis, (2) mitotic activity [measurement of PHH3 antibodies], and (3) measurement of HIF-1*α* expression.

**Results:**

The incidence of lung nodules was significantly increased in CS. CS exposure significantly increased PHH3 and HIF-1*α* expression. PJ supplementation attenuated the formation of lung nodules and reduced PHH3 and HIF-1*α* expression.

**Conclusion:**

PJ supplementation significantly decreased the incidence of lung cancer, secondary to CS, prevented the formation of lung nodules, and reduced mitotic activity and HIF-1*α* expression in an animal model.

## 1. Introduction

Lung cancer constitutes more than a sixth of all cancer deaths worldwide and is the leading cause of cancer death in the United States for both men and women [1, 2]. In 2014, chronic lower respiratory disease (COPD) was the third leading cause of death in the United States after heart disease and cancer [1]. Most cases of lung cancer and COPD are associated with combustible tobacco smoke, and cigarette smoke (CS) remains the basic vehicle for delivering combustible tobacco smoke leading to the development of lung cancer and COPD [3, 4]. COPD is also an independent risk factor for lung cancer among smokers, and the probability of lung cancer increases with the increasing severity of COPD [5, 6]. The pathophysiology of lung cancer and COPD shares similar pathways that lead to cellular injury, DNA damage, and mutations [7].

Oxidative stress (OS) is a common pathway in the pathogenesis of COPD and lung cancer. Previously, we reported higher OS in animals exposed to CS. Emphysematous changes and destruction of lung architecture were observed with three-month CS exposure as well [8]. CS contains free radicals and reactive oxygen species (ROS) that deplete extracellular and intracellular antioxidants causing oxidative damage to DNA, proteins, and lipids [9, 10]. Antioxidant depletion, induced by CS, correlates with carcinogenesis of lungs and other organs as well [11]. DNA is particularly vulnerable to OS, and DNA repair may result in mutations leading to the development of malignant cells [12, 13].

Avoidance of CS exposure and quitting smoking remain the key and most effective strategy that will prevent OS and tissue injury. An alternative strategy, however, is to strengthen the defense mechanisms against carcinogenesis of CS by exogenous supplementation of pharmacological and dietary agents. This approach, referred to as “chemoprevention,” is currently utilized in the fight against cancer and other human disorders such as cardiovascular diseases [14, 15]. Chemoprevention supplementation that will result in significant reduction in OS may prevent tissue injury and eventually may reduce the incidence of lung cancer secondary to CS exposure.

*Punica granatum* L. (Punicaceae) is very rich in polyphenols and other biologically active compounds like flavonoids, gallic acid, ellagic acid, and ellagitannins. The combination of polyphenols with other phytochemicals such as ellagic acid synergistically augments the superior antioxidant properties of PJ [16, 17]. Polyphenols act as oxygen radical and hydroxyl radical scavengers and increase antioxidant glutathione levels by inducing glutamate cysteine ligase expression. As an anti-inflammatory agent, polyphenols inhibit nuclear factor kappa-light-chain-enhancer of activated B cells (NF-*κ*B) and interleukin 8 (IL-8) release in lung cells [18, 19]. Recent studies have demonstrated that pomegranate extracts selectively inhibit the growth of breast, prostate, colon, and lung cancer cells in vitro [19, 20].

This study examined whether the administration of pomegranate juice (PJ) attenuates the expression of tumor markers of lung cancer and decreases the incidence of lung cancer secondary to CS in an animal model.

## 2. Methods

The study was approved by the Institutional Animal Care and Use Committee. Two-month-old adult male AJ mice (22–25 g body weight) were subjected to a 12-hour dark/light cycle. Room and chambers' temperature were maintained at 22–24°C. Animals were allowed unlimited access to water and standard rodent chow except when they were placed in the exposure apparatus. CS exposure apparatus (ONARES, CH Technologies, USA) consisted of a smoke generator, mixing/conditioning chamber, and a twelve “nose only” rodent exposure carousel. One port of the carousel was dedicated for sampling analysis, and the remaining 11 ports were used for animal exposure [8]. Animals were divided into 4 groups: control, CS, CS + PJ, and PJ. Each group consisted of 11 animals, and they were acclimated to retainers for one week prior to initiating room air or CS exposure. Mice were then positioned in retainers and placed into the holes of the carousel. Animals received a continuous flow of CS or room air into the airways via the “nose only” delivery system. As described before, CS was generated from 3R4F cigarettes (University of Kentucky, Lexington, KY) with 0.9 mg total particulate matter (TPM), 9.4 mg tar, and 0.726 mg nicotine per cigarette. The machine was set at one puff every minute with duration of two seconds per puff and a volume of 35 ml per puff [8].

CS or room air exposure was set for 4 hours per day (2 hours in the morning and 2 hours in the afternoon), five days per week, for 5 months. Animals were then housed for additional 4 months, with no CS exposure, to allow for tumor development [21]. CS + PJ and PJ groups, however, continued to receive PJ supplementation for the additional waiting period of 4 months. At the conclusion of the experiment, animals were anesthetized and then exsanguinated by severing the aorta. The diaphragm was dissected to allow free lung expansion.

### 2.1. Pomegranate (PJ) Processing and Administration

PJ concentrate (Wonderful variety, POM Wonderful, Los Angeles, USA) was administered via drinking bottle to the CS + PJ and PJ groups. Starting one week prior to CS or room exposure, PJ was initiated and maintained throughout the experiment. Animals received 80 *μ*mol/kg/day of PJ while the control and CS received free water. The dose of PJ supplementation was deduced from previous studies [22].

### 2.2. Histopathology

All lung lobes were fixed in buffered formalin and embedded in paraffin. A series of 10 (5 *μ*m) sections were cut systematically from the right and left lung lobes and placed into Eppendorf tubes. Sections 1 and 5 from each series were placed on slides, stained with H&E, and evaluated by light microscopy for cellular hyperplasia, adenoma, or adenocarcinoma. Further sectioning was continued in a series of 10 thin (5 *μ*m) sections at a time throughout the available paraffin blocks of lung tissues. In areas of the lung where tumor was observed on light microscopy, the remaining associated 5 *μ*m thick sections were de-paraffinized and utilized for further analysis.

### 2.3. Tumor Nodule Enumeration

Tumors were identified by light microscopy and statistically evaluated in terms of multiplicity (mean number of tumors per animal over the total number of animals), incidence (number of animals with tumors over the total number of animals), and tumor size (in mm). The diameter of tumor nodule was measured for each nodular tissue based on random fields observed at a total magnification of ×200 using a metric ruler, and the tumor size was calculated as reported previously [22].

### 2.4. Immunofluorescence Analysis for Cellular Proliferation and Proangiogenesis Factors

Where tumor was observed, contiguous 5 *μ*m thick sections were de-paraffinized in xylene, rehydrated in ethanol, and then rinsed briefly in deionized water. Recovered sections were heated in citrate buffer at 74°C (BioGenex, USA) for 40 min. The antigen-retrieval slides were allowed to cool at room temperature for 30 min and rinsed twice in deionized water following washing in phosphate-buffered saline (PBS) for 5 min.

#### 2.4.1. Anti-Phosphohistone-H3 (PHH3) Antibody Expression

Increased pHH3 levels are associated with increased mitotic activity and can be used as an indicator of malignancy [23]. Tissue sections were incubated in 3% hydrogen peroxide for 30 min to block endogenous peroxidases, rinsed once in deionized H_2_O, and followed with three times in PBS. Sections were then incubated with 3% normal goat serum in PBS for 1 hour to block nonspecific binding proteins. Retrieved sections were placed overnight at 4°C in a moist chamber with rabbit polyclonal anti-PHH3 at a dilution 1 : 500 (Millipore, Billerica, MA, USA). After overnight incubation, slides were rinsed twice in PBS (10 min each) and incubated with goat anti-rabbit fluorescence antibodies at 1 : 500 dilutions (F2765, Invitrogen Molecular Probes, Carlsbad, CA) for 1 hour. Slides were rinsed twice in PBS (10 min each), and nuclei were then distinguished by counterstaining with Hoechst 33258 stain (Sigma-Aldrich, Darmstadt, Germany).

#### 2.4.2. Hypoxia-Inducible Factor-1*α* Expression

Hypoxia-inducible factor 1*α* (HIF-1*α*) expression is significantly associated with cellular proliferation, angiogenesis, and lung cancer [24]. To evaluate HIF-1*α* levels, tissue sections were incubated with rabbit polyclonal HIF-1*α* antibodies at a dilution 1 : 50 in 1% normal goat serum (Santa Cruz Biotechnology Inc., CA, USA). Hypoxic lung tissues from previous animal experiments were utilized for positive control, and fluorescent images were scanned for signal with the laser scanning confocal microscope (LSM 710, Zeiss, Germany) [19].

## 3. Results

### 3.1. Histopathology and Lung Nodule Enumeration

**Figure 1:**
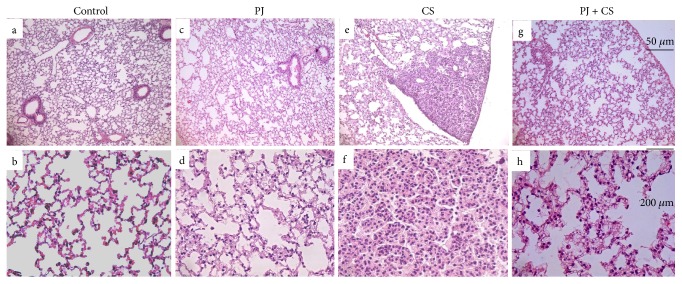
H&E examination under light microscopy of lung tissues from the control (a, b), PJ (c, d), CS (e, f), and PJ + CS (g, h). Note: normal lung architecture observed in lung tissue from control showing, at lower magnification (original magnification: ×20), thin interstitial alveolar wall and capillary vessels (a). At higher magnification (original magnification: ×40), normal lung tissue is shown and rare inflammatory cells are noted (b). Similar findings were observed in the PJ-only group (c, d) and PJ (b). (e) A pulmonary nodule noted with CS exposure and at a higher magnification (original magnification: ×40), the nodule consists of round epithelial cells with high nuclear-cytoplasm ratio grouping together and forming characteristic acini. The CS + PJ revealed no lung nodules and minimal injury and damage to the lung parenchyma and preservation of pulmonary alveoli (g, h). CS: cigarette smoke; H&E: hematoxylin and eosin; PJ: pomegranate juice.

**Figure 2:**
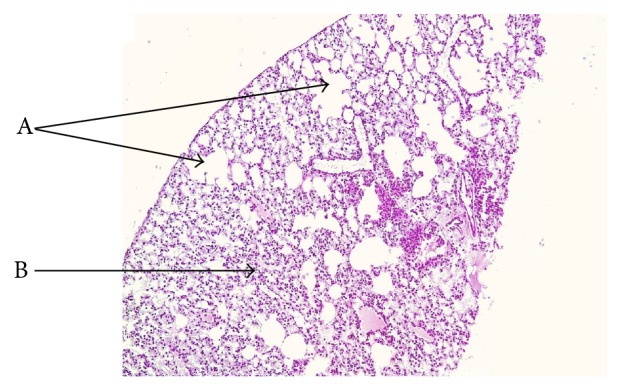
H&E examination under light microscopy of lung tissues from CS animals. Note: CS mice showing major elements of lung injury secondary to CS exposure. Arrows are pointing to the destruction of alveolar walls (A) and infiltration of inflammatory cells (B).

Control (Figures 1(a) and 1(b)) and PJ (Figures 1(c) and 1(d)) groups revealed normal alveolar structure with minimal infiltrates of inflammatory cells and thin alveolar walls. In contrast, lung tissues of the CS-exposed mice showed damaged alveolar architecture, emphysema, and higher levels of inflammatory cells (Figure 2, A). In addition, a significant number of lung nodules were identified in CS-exposed mice (*P* < 0.05 when compared to control) (Table 1). Observed lung nodules in the CS group consisted of atypical round or oval epithelial cells, containing hyperchromatic nuclei and forming characteristic glands or acini (Figures 1(d) and 1(e)). PJ supplementation to CS animals attenuated the findings noted in CS animals; normal alveolar structure with minimal emphysematous changes and no pulmonary nodules were observed (Figures 1(g) and 1(f)).

### 3.2. Mitotic Activity Assessment

**Figure 3:**
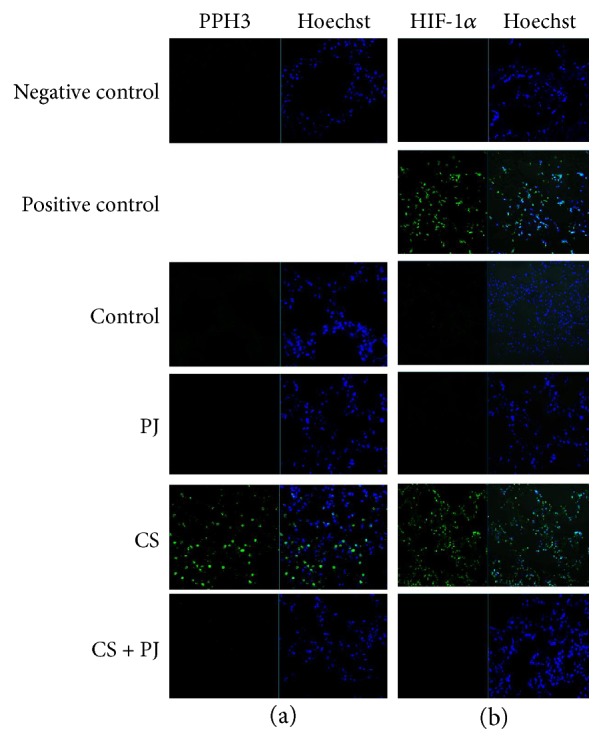
CS-induced increased mitotic activity PHH3 (a) and hypoxia-inducible factor 1*α* expression (a). (a) All images were captured using 20x objective. 5 *μ*m thick sections were mounted on microscope slides and incubated with polyclonal anti-PHH3. Significant increase in PHH3 expression was observed in the lungs of CS animals suggesting increased mitotic activity. PJ supplementation prevented CS-induced PHH3 expression. (B) All images were captured using 20x objective. 5 *μ*m thick sections were mounted on microscope slides and incubated with rabbit polyclonal HIF-1*α* antibodies. Significant HIF-1*α* expression was observed in CS when compared to control, and PJ supplementation prevented CS-induced HIF-1*α* expression. CS: cigarette smoke; HIF-1*α*: hypoxia-inducible factor 1*α*; PJ: pomegranate juice. PHH3: phosphohistone-H3.

PHH3 is a mitosis-specific marker and a reliable indicator of mitotic activity in different types of tumors [23, 25]. There was no difference in PHH3 activity between control and PJ. In CS animals where pulmonary nodules were detected, a significant increase in PHH3 expression was observed (Figure 3(a)). PJ supplementation to CS animals attenuated PHH3 expression to values comparable to the control.

### 3.3. Hypoxia-Inducible Factor-1*α* (HIF-1*α*) Expression

HIF-1*α* plays a central role in tumor progression by activating target genes that are associated with oxygen homeostasis and angiogenesis [24]. Because hypoxia is associated with increased HIF-1*α* expression, positive control for HIF-1*α* was confirmed in mouse hypoxic lung tissues (Figure 3(b)). HIF-1*α* activity was then evaluated in all animal groups. A significant increase in HIF-1*α* activity was noted in lung tissues of CS animals where lung nodules were detected. The activity of HIF-1*α* in the PJ or in CS + PJ groups and their HIF-1*α* expression values were essentially comparable to the control.

## 4. Discussion

Previously, we showed that OS secondary to CS is associated with alveolar destruction and lung injury [8]. In this study, we documented the beneficial effects of antioxidant supplementation, represented by PJ, in preventing the formation of CS-induced lung nodules and in attenuating markers associated with lung cancer in a chronic CS animal model. Antitumorigenic effects of polyphenols, an integral component of PJ, fruits, and vegetables, were reported in previous animal studies. Balansky et al. reported the inhibition of lung nodule development by berry extracts in mice exposed to CS extract, and Bao et al. reported that apple polyphenol protected against CS-induced acute lung injury [26, 27]. Other studies examining the effects of polyphenols and antioxidants utilized known lung carcinogens rather than chronic CS exposure in animal models [28]. The chemo-preventive ability of PJ is through modulating multiple signaling pathways. PJ increases p21 and p27 expression, as both proteins are major mediators of p53-dependent cell cycle arrest in response to DNA damage [29]. PJ inhibits MAPK (mitogen-activated protein kinase) and NF-*κ*B signaling pathways. Increased activities of these pathways are associated with cellular proliferation and malignancy [30]. Finally, PJ prevents mutagenic activities by inhibiting benzo[a]pyrene (abundant in CS)-induced DNA adduct formation and decreases markers of angiogenesis and cellular proliferation as well [29, 31].

The effects of nutritional elements coupled with human habits may lead scientists to design new innovative strategies aimed at reducing the incidence of lung cancer [32]. In Shanghai for example, women who are nonsmokers and drink green tea on a regular basis are 35% less likely to develop lung cancer when compared to women who are not regular green tea drinkers. In a population-based case study, Christensen et al. demonstrated that lower intake of dietary flavonoids is associated with an increase in incidence of cancer [33, 34]. Not all chemo-preventive studies were successful; beta-carotene supplementation was tested in several controlled trials and offered no beneficial effects [35]. On the contrary, additional harmful effects were observed in smokers who received high doses of beta-carotene suggesting that beta-carotene may act as a prooxidant when administered in high doses. Distorting the delicate balance of achieving adequate but not excessive supplementation of antioxidants is crucial to elicit protective effects aimed at attenuating oxidative stress secondary to CS and thus preventing relentlessly the associated inflammation and the evolution of lung cancer.

The design of this study insured chronic CS exposure through the nose-only delivery system which delivered CS and not CS extract in a precise and predictable manner. The duration of the study was five months of CS exposure and four months of waiting period thus providing ample time for the development of lung nodules and lung cancer [21]. The waiting period of four months may mimic the status of ex-smokers who quit smoking and yet continue to be at a higher risk for developing lung cancer. This study suggests that continued supplementation of antioxidants may be an innovative modality to combat the development of lung cancer in smokers and in ex-smokers as well. However, additional animal and human studies to define the perfect antioxidant and, more importantly, determine an adequate but not excessive dosage needed to reduce the incidence of lung cancer secondary to combustible tobacco smoke exposure are required.

## Figures and Tables

**Table 1:** Incidence of lung nodules and tumor multiplicity in CS animals identified by light microscopy.

	Control	PJ	CS	PJ + CS
Total number of animals	11	11	11	11
Number of animals with lung nodules	0	0	4	0
Mean number of lung nodules per animal	—	—	1.30 ± 0.25	—
Tumor multiplicity (mean number of lung nodules per animal/nontumor-bearing animals)	—	—	0.19	—
Tumor incidence (number of animals with lung nodules/total number of animals)	—	—	0.36	—
Mean nodular size (mm)	—	—	1.20 ± 0.29	—

Note: PJ supplementation in the CS + PJ group prevented the formation of lung nodules that were noted in CS. CS: cigarette smoke; PJ: pomegranate juice.
